# An Interesting Care of Hemiplegia

**Published:** 1865-11

**Authors:** Morton M. Eaton

**Affiliations:** Peroia, Ill.


### AN INTERESTING CASE OF HEMIPLEGIA.

By MORTON M. EATON, M. D., of Peroia, Ill.

Was called about two weeks since to see the daughter of Mr. M., of this city, aged 14 years.

Found her suffering from Hemiplegia. The left leg and arm were insensible to pain and motion extremely difficult, seemingly from want of power. The face wore a natural expression, the tongue inclined to the left side. The pulse was natural on the right side; the affected members were cold, while the other parts of the body were of a normal temperature. The tongue was clean ; respiration natural.

My patient was able to converse readily, and from her and her mother I learned that she had never menstruated; that this attack had been coming on for three days, beginning with numbness; that about two months before she had been troubled with a numbness in these members, and a pain in the back and thighs; that the bowels were regular; the appetite was impaired. I examined the spine carefully, but found no indications of any abnormal condition; the brain also seemed in healthy operation.

Having found no evidences of disease, and from the fact of her never having menstruated, I examined the hypogastric region and found much tenderness, and passing my finger between the labia and finding no hymen, I proceeded to pass my finger internally, which caused no pain till I came in contact with the os uteri, when I found that the womb was much inflamed, and extremely sensitive. This being the state of affairs, I determined to direct my attention mainly to this condition of the womb. I applied warm fomentations of hops over the hypogastric region, wrapped the affected limbs in flannel, and made the following prescription: ℞.—Pill Hydrarg. gr. x; Ferri Pulv. gr. v; Rhei Pulv. gr. x; Quin. Sul. gr. v; Strychnia Nit. grss.—M. Pill No. xv., div. S.—Take one three times a day.

Under this treatment she rapidly improved, the tenderness and congestion disappearing; the power of motion and feeling returned within a week, and the limbs have regained their natural temperature.

The case was new to me because of its occurring in the virgin. I have consulted those authors that were at hand, and I find no cases of this recorded, though Dr. Churchill, in treatise on Diseases of Women, page 732, states that "A nervous or hysterical paralysis may occur occasionally in the unimpregnated state."

Dr. Simpson says Albuminuria in the impregnated state sometimes gives rise to local paralysis, functional lesions, &c.

Dr. Romberg says, in his work published by the "Sydenham Society," that paralytic attacks, arising from morbid conditions of the sexual system, through reflex influence upon the spinal cord, affect both sides.

I hope if other physicians have had similar cases, or can better explain the cause or the symptoms, than that the retention or non-appearance of the menses had caused an engorged and congested state of the uterus, and that this engorgement, by some means acting by means of reflex action on the spinal cord, caused the coldness, insensibility and loss of motion, we may hear their theory. I can see but one other way of explaining the case, that is, that we are aware that the suppression of the menstrual secretion frequently causes congestions of distant organs, and that possibly there was present a fullness of the spinal arterial twigs of sufficient magnitude to cause pressure on the side of the spinal cord. I shall recommend for the next too weeks, warm hip baths and the administration of small doses of Iron, Aloes and Myrrh, thinking that if the menstrual functions were fully established, she would have no return of these troubles, and fearing and expecting that if it is not established, that she may be attacked again as before in one or two months.

